# Efficacy of Low-Molecular-Weight Heparin in the Treatment of Severe Acute Pancreatitis: A Comparative Study at a Tertiary Health Center

**DOI:** 10.7759/cureus.65736

**Published:** 2024-07-30

**Authors:** Shashirekha CA, Kavitha Gondesi, Krishna Prasad K, Sai Vikram Yarremsetty

**Affiliations:** 1 General Surgery, Sri Devaraj Urs Medical College, Kolar, IND

**Keywords:** low-molecular-weight heparin, microcirculatory disturbance, microthrombosis, inflammatory response, severe acute pancreatitis

## Abstract

Introduction

Acute pancreatitis is caused by multiple factors. The disease can progress to severe acute pancreatitis (SAP) rapidly, which is a fatal condition. SAP is associated with systemic inflammatory response and disturbances in microcirculation, which are responsible for the high rate of mortality. Low-molecular-weight heparin (LMWH), apart from preventing thrombus formation, also has a property of reducing the release of cytokines and inflammatory mediators. This aids in the improvement of microcirculation of the pancreas.

Materials and methods

A prospective hospital-based study was conducted on 60 patients diagnosed with SAP. Patients were randomly divided into two groups: conventional treatment group (C group consisting of 30 patients) and LMWH in addition to conventional treatment (L group, consisting of 30 patients). Clinical and laboratory parameters and computed tomography (CT) scores of pancreatic necrosis (CTSPN) were compared in the two study groups.

Results

No significant difference (p > 0.05) was observed in the clinical and laboratory parameters and CTSPN among the two study groups at the time of admission. On initiation of treatment, the rate of improvement in symptoms and laboratory parameters were significantly higher in the L group compared with the C group (p < 0.05). Acute physiology and chronic health evaluation (APACHE) II score and development of complications were significantly lower in the L group compared with the C group (p< 0.05). CTSPN was found to be very low in the L group compared with the C group (p < 0.05). After two weeks of treatment, the recovery rate in response to treatment was observed to be higher in the L group compared to the C group, and the mortality rate was very low in the L group (p <0.05, significant).

Conclusion

The addition of LMWH to conventional treatment in SAP augments the effect and improves the clinical response, improving the recovery rate and decreasing mortality. LMWH is highly effective for the treatment of SAP, with a good safety profile and easy availability and without high financial burden.

## Introduction

Acute pancreatitis can be seen commonly with wide variations in severity. Mild acute pancreatitis will be mostly uneventful, and the majority of patients recover spontaneously within a week. Moderately severe pancreatitis and severe acute pancreatitis are associated with complications, either local or local with systemic involvement. The development of severe acute pancreatitis can be rapid. Severe acute pancreatitis is generally associated with widespread systemic involvement, making it a lethal condition. Systemic inflammatory cascade and microcirculatory disturbances are seen in almost all cases of severe acute pancreatitis [1].

Renzuli et al. [1] stated that systemic inflammatory cascade and microcirculatory disturbances play a key role in the development of severe acute pancreatitis and are responsible for multiple organ systemic failure and high mortality of 20-40% in sepsis-induced pancreatic necrosis.

Karne et al. [2] in his study stated that multiple factors and causes are responsible for acute pancreatitis. Chief pathology is the effect on the acinar cells of the pancreas. Insult to acinar cells results in premature activation and retention of proteolytic pancreatic enzymes. Inflammatory activity in its early stages involves the activation of neutrophils, macrophages, and endothelial cells. The release of proinflammatory cytokines along with higher levels of inflammatory factors results in the development of a potent inflammatory response, which is responsible for microvascular disturbances leading to hemorrhagic necrosis. At the cellular and tissue level, ischemic insult occurs, followed by reperfusion causing ischemia-reperfusion injury, which ultimately leads to the formation of microthrombi. These cellular disturbances evoke the secretion of cytokines. A high load of inflammatory mediators causes local insult and widespread systemic complications [1,2].

Pathogenesis of severe acute pancreatitis includes premature activation of pancreatic proteases (digestive enzymes) and their extravasation into the pancreas and surrounding peripancreatic tissue. Excessive leukocyte activation results in the release of cytokines and other inflammatory mediators. These inflammatory mediators in high quantities stimulate an inflammatory cascade, resulting in systemic inflammatory response syndrome (SIRS) [3]. Local and widespread systemic infection, necrosis of pancreas and surrounding tissue, and development of organ failure carry poor prognosis. Pancreatic necrosis development and its pathogenesis affect other organs, including the lungs, liver, and intestine, in the due course of severe acute pancreatitis [3].

Studies by Dobosz et al. [4] observed that low-molecular-weight heparin (LMWH) reduces the release of cytokines and inflammatory mediators in the disease course. TNF-α (tumor necrosis factor-α, an inflammatory mediator) production is decreased by LMWH, preventing the development of inflammatory storm. LMWH has anti-thrombin activity. LMWH can decrease the release of endothelin-1 (ET-1) resulting in an improved microcirculatory environment, and the anti-thrombus effect reduces microthrombi development. These factors are beneficial in the improvement of the microcirculation of the pancreas. The above-stated findings demonstrate the therapeutic effect of LMWH in the treatment of severe acute pancreatitis.

A study by Qiu et al. [5] in a rodent model identified that LMWH can effectively improve survival rate in severe acute pancreatitis involving rodents. A study by Balthazar et al. [6] about assessing the severity of pancreatitis stated that computed tomography (CT) scan helps in an early detection rate of 90%, almost with 100% sensitivity after four days for pancreatic necrosis. CT severity index has shown an excellent correlation with the development of local complications and the incidence of death in this population.

The objective is to study the effect of LMWH in addition to conventional treatment in decreasing the APACHE II score by its impact on microcirculatory disturbances and observe the improvement of clinical parameters in patients with severe acute pancreatitis. The aim of this study is to determine the potential benefits and risks associated with LMWH therapy, by assessment of laboratory parameters, coagulation profiles, and clinical outcomes. This study aids in improved therapeutic strategies for severe acute pancreatitis, providing better options for improving the recovery rate, decreasing the mortality rate, and improving patient care.

## Materials and methods

Study design, sample size, and source of data

A prospective hospital-based study was carried out on 60 participants, allotted into two groups of 30 each using the odd/ even method.

The two groups, those receiving conventional therapy alone and those receiving conventional therapy and LMWH, were studied during their hospital course in the Department of General Surgery at Sri Devaraj Urs Medical College (SDUMC) over a period of six months after obtaining ethical committee approval from the Institutional Ethics Committee (IEC) (ref. no. SDUMC/KLR/IEC/606(A)2023-2024).

Inclusion and exclusion criteria

Patients diagnosed with severe acute pancreatitis and having pancreatic necrosis and/or dysfunction of one or more organs, patients with laboratory values of serum amylase levels three times higher than the normal values, and patients presenting with acute physiology and chronic health evaluation score (APACHE) II more than or equal to eight at the time of admission were included in the study. Patients less than 12 years of age, patients with known allergy to LMWH, pregnant females and lactating mothers, patients with known coagulation disturbances or coagulopathies, and patients who are already undergoing hemodialysis are excluded from the study.

Method of data collection

After obtaining approval from the IEC and informed written consent from the study participants or legal guardians, the study was conducted in the Department of General Surgery on 60 patients admitted and diagnosed with severe acute pancreatitis from October 2023 to March 2024 at R L Jalappa Hospital (RLJH), Kolar.

All patients were evaluated with all pertinent laboratory values. All the demographic details were collected from the participants.

C Group (Conventional Treatment)

Among 30 patients enrolled, 18 were males and 12 were females, with age between 15 and 72 years.

At the time of admission, all 30 patients (100%) had severe abdominal pain, 25 patients (83.3%) had nausea and/or vomiting, and 19 patients (63.3%) had fever. One (3.3%) patient presented with acute respiratory distress syndrome (ARDS), one patient (3.3%) was in shock, two (6.6%) patients were diagnosed with acute renal failure (ARF), one (3.3%) patient had mild upper gastrointestinal (GI) bleeding, and one was diagnosed with disseminated intravascular coagulation (DIC). One patient (3.3%) had a failure of two or more organs. The average APACHE II score among the 30 patients was 11.5 ± 3.4.

L Group (LMWH + Conventional Treatment)

Out of 30 patients, 22 were males and eight were females, aged between 16 and 75 years.

At the time of admission, all 30 patients (100%) had severe abdominal pain, 25 patients (83.3%) had nausea and/or vomiting, and 19 (63.3%) patients had fever.

Among 30 patients, three (10%) patients had ARDS, one (3.3%) patient was in shock, two (6.6%) patients were diagnosed with ARF, one (3.3%) patient had mild upper GI bleeding, and one (3.3%) patient had a failure of two or more organs. The average APACHE II score in the 30 patients was 11.6 ± 3.6.

At the time of admission, clinical parameters and APACHE II scores between the C group and L group were not significantly different (p > 0.05).

Treatment protocol

C Group

Conventional treatment includes nil per oral status (fasting), fluid and electrolyte balance maintenance, gastrointestinal decompression, management of shock, pancreatic enzyme inhibitor (somatostatin), proton pump inhibitors (PPIs), antibiotics, and symptomatic treatment (analgesics and antiemetics).

L Group

LMWH (injection enoxaparin) was added at a dose of 0.1 mg/kg once a day by a subcutaneous route from the day of admission for seven days to the conventional treatment given in the C group.

Statistical analysis

Data were entered into a Microsoft Excel data sheet (Microsoft Corporation, USA) and were analyzed using IBM SPSS Statistics for Windows, Version 22.0 (released 2013, IBM Corp., Armonk, NY). Categorical data were represented in the form of frequencies and proportions. Continuous data were represented as mean and standard deviation. The normality of the continuous data was tested by the Shapiro-Wilk test. Student's t-test and repeated-measure ANOVA tests were used to compare continuous variables. The chi-square test was used to compare categorical variables. For the graphical representation of data, MS Excel and MS Word were used to obtain various types of graphs, such as bar diagrams and pie diagrams. A p-value (probability that the result is true) of <0.05 was considered statistically significant after assuming all the rules of statistical tests.

Observed variables

Clinical Parameters

Improvement rate in symptoms, APACHE II score, development and cure of complications, development of organ failure and recovery, mortality rate in hospital, cure rate, and average hospital stay were observed and compared.

Laboratory Tests

Laboratory investigations compared in the two groups include white blood cell count (WBC), hematocrit (HCT), platelet count (PLT), serum amylase levels (Sr amylase), coagulation parameters prothrombin time (PT), fibrinogen (FBI), blood sugar, serum calcium levels, liver function tests (LFT), renal function tests (RFT), and arterial blood gas (ABG) analysis. The above-mentioned Investigations were measured and compared between two groups at the time of admission, one week after treatment, and two weeks after treatment.

CT Scan Scores

CT score of pancreatic necrosis (CTSPN) was measured and compared between the two groups at the time of admission, after one week of treatment, and after two weeks of treatment.

## Results

Among 30 patients enrolled, 18 were males and 12 were females in C group. In L group out of 30 patients, 22 were males and 8 were females(Table 1).

**Table 1:** Gender distribution in the two groups C = conventional treatment, L = low-molecular-weight heparin (LMWH) added to the conventional treatment group

Group	Male	Female	Total
C	18	12	30
L	22	8	30

Clinical symptom improvement rate

In the L group, the symptom improvement rate was high after one week in 17 patients (56.7%) and after two weeks of treatment in 28 patients (93.3%). The C group showed lower rates of symptom improvement: eight patients (26.7%) after one week and 21 patients (70%) after two weeks of treatment. The symptom improvement rate in the L group was observed to be higher on comparison with the C group and is statistically significant (p < 0.05).

Cure rate and mortality rate

The cure rate in the C group(69.4%) was lower than in the L group (89.6%, 26/30) and is statistically significant (p < 0.05). Mortality in the C group (30.6%, 9/30) was higher than mortality in the L group (10.4%, 14/30), which is statistically significant(p < 0.05) (Table 2).

**Table 2:** Cure rate, mortality rates, and duration of hospital stay in the two groups C = conventional treatment, L = low-molecular-weight heparin (LMWH) added to the conventional treatment group

Group	Cure rate	Mortality rate	Hospital stay
C	70%	30%	18 ± 3 days
L	90%	10%	14 ± 2 days

APACHE II scores

APACHE II scores were not significantly different between the two groups at the time of admission: 11.6 ± 3.6 in the C group and 11.5 ±3.4 in the L group. No significant difference after one week of treatment was observed in the two groups: 10.4 ± 2.9 in the C group and 10.5 ± 2.3 in the L group (p > 0.05). Significant difference was seen after two weeks of treatment in the APACHE II scores between the two groups: 8.5 ± 1.8 in the L group, which was significantly lower than that in the C group, i.e., 9.6±2.4 (p < 0.05)(Table 3).

**Table 3:** Serum amylase, CTSPN score, and APACHE II score at admission and after one week and two weeks of treatment CTSPN = computed tomography scan score of pancreatic necrosis, APACHE = acute physiology and chronic health evaluation

Group	Time point	Serum amylase	CTSPN	APACHE II
C group	Admission	368.4±89	5 ± 1.5	11 .5±3.4
	One week after treatment	316.3±86	4.9 ± 2.4	10.5±2.3
	Two weeks after treatment	173.8±34	4.3±2.6	9.6±2.4
L group	Admission	362.9±96	5.4 ± 1.9	5.4±1.9
	One week after treatment	119.33±32	3.8 ± 2.2	3.8±2.2
	Two weeks after treatment	91.3±281	2.1 ± 1.0	2.1±1

CTSPN

At the time of admission, the CTSPN score in the L group was 5.4 ± 1.9 and in the C group was 5.0 ± 1.5, which were not significantly different (p > 0.05). The CTSPN score in the L group was 3.8 ± 2.2 after one week of treatment and 2.1 ± 1.0 after two weeks of treatment. The CTSPN score in the C group after one week of treatment was 4.9 ± 2.4 and 4.3± 2.6 after two weeks of treatment. Scores were very low in the L group when compared to the C group after two weeks of treatment, which is statistically significant (p < 0.05) (Table 3).

Serum amylase

At the time of admission, the serum amylase levels were not significantly different between two groups (p > 0.05). After one and two weeks of treatment, the serum amylase levels were significantly lower in the L group compared with the C group (p < 0.05) (Table 3).

PT, fibrinogen, and platelet count

No difference was found in the blood results of PT and platelet count at the time of admission and after one and two weeks of treatment between two groups (p > 0.05), which are statistically insignificant (Table 4).

**Table 4:** Coagulation parameters at various points of time PT = prothrombin time, FBI = fibrinogen, PLT = platelet count

Group	Time point	PT (seconds)	FB (g/L)	PLT (x 10^9^/L)
C group	On admission	15.0 ± 1.5	3.5 ± 0.7	180 ± 27
	One week after treatment	15.0 ± 2.5	3.0 ± 0.6	240 ±27
	Two weeks after treatment	15.0 ± 2.5	2.5 ± 0.5	292 ± 37
L group	On admission	15.0 ± 1.8	3.6 ± 0.9	175 ± 19
	One week after treatment	15.0 ± 1.9	3.1 ± 0.7	230 ± 28
	Two weeks after treatment	16.0 ± 1.6	3.0 ± 0.6	294 ± 49

After two weeks of treatment, fibrinogen values decreased drastically in the C group, but no significant change was observed in the L group (Table 4). There was no significant coagulation disturbance in both groups.

Multiple organ dysfunction syndrome (MODS)

Multiple organ systems were involved in both groups in the treatment period. During the treatment period, in the C group, ARDS developed in 10 patients (34.4%), PE in three patients (10.0%), and GI bleeding in one patient (3.4%). Among them, seven patients (24.1%) developed failure of two or more organs.

In the L group, ARDS developed in three patients (11.1%), PE in one (3.3%) patient, and GI bleeding in one patient (3.4%). Among them, two patients (6.8%) developed failure of two or more organs (Table 5).

**Table 5:** Number of patients affected with various complications and successful treatment rate ARDS = acute respiratory distress syndrome, ARF = acute renal failure, PE = pancreatic encephalopathy, GI = gastrointestinal, MODS = multiple organ dysfunction syndrome

Complication	Group	On admission (number of cases)	During treatment (number of cases)	Successful treatment rate (percentage)
ARDS	C	1	10	72 %
	L	3	3	83.3%
Shock	C	1	0	100 %
	L	1	0	100%
ARF	C	2	0	100 %
	L	1	0	100%
PE	C	0	3	66.6 %
	L	0	1	100%
Gastrointestinal bleeding	C	1	1	100 %
	L	1	1	100%
MODS	C	1	7	37.5 %
	L	1	2	66.6%

Development of organ failure, more frequently ARDS, was seen in the C group. The rate of treatment success was lower in the C group compared to the L group( Table 5).

Average hospital stay

The average hospital stay in the C group was 18 ± 3 days, and in the L group, it was 14 ± 2 days.

## Discussion

Studies were conducted focusing on the effect of LMWH therapy in preventing microvascular damage, which prevents and attenuates organ-level damage at the pancreas, kidneys, lungs, and brain. Organ damage can be prevented by LMWH therapy, by attenuation of ET-I, inhibiting NF-kB, which decreases IL-6 and TNF-α. Treatment with LMWH prevents and treats microthrombosis, resulting in improved pancreatic microcirculation and systemic microcirculation at the lung, kidney, and brain, aiding in decreased mortality [7].

Widespread systemic inflammation finally leads to the development of MODS [7]. MODS is a major reason for mortality in patients with severe acute pancreatitis. Our study showed a consistent relation between the development of MODS and mortality in severe acute pancreatitis. Inhibition of the activity of cytokines in inciting inflammatory response, correcting the microcirculatory disturbances in the pancreas, is the pivotal step in the treatment of severe acute pancreatitis.

The blood supply of the pancreas is by the lobar artery, which is an end artery without any branches. In case of obstruction of the lobar artery, pancreatic lobules will be devoid of blood supply, resulting in sublobular ischemia and finally necrosis. Evidence-based studies show the relationship between microvascular disturbances locally(at the pancreas) and systemically. Pathogenesis involves vasoconstriction in involved areas, shunting, defective perfusion, and raised blood viscosity leading to increased coagulation. Increased coagulation is the chief pathology involved in the progression to severe acute pancreatitis. Oxygen-derived free radicals released due to ischemia-reperfusion injury exacerbate the above-said microvascular disturbances [8,9]. Pathogenesis can be halted by preventing and treating microvascular disturbances locally (at the the pancreas) and at the systemic level. Improvement in microvasculature prevents progression to severe acute pancreatitis and development of multi-organ dysfunction.

Leizorovicz et al. conducted a study [10] comparing the efficacy and safety of LMWH with unfractionated heparin in patients with deep venous thrombosis. LMWH has a higher benefit-to-risk ratio compared to unfractionated heparin for venous thrombosis treatment. Our study also showed very minimal coagulation abnormalities like bleeding, indicating that LMWH is safe.

The results of our study showed that the clinical improvement rate was significantly higher in the L group than the C group, and the development of complications like ARDS, multiple organ dysfunction, mortality, and average hospital stay was lower in the L group than in the C group. These results suggest a significant effect of LMWH in the treatment of severe acute pancreatitis.

Limitations of the study

This study was conducted on a small sample size and at a single institution. Conducting a large-scale study on a larger sample size will be helpful in validating the results of LMWH use in the treatment of severe acute pancreatitis.

## Conclusions

LMWH effectively improves recovery, prevents severity progression and organ failure, and decreases mortality in severe acute pancreatitis. The good safety profile of LMWH makes it a better and safer option in severe acute pancreatitis treatment. LMWH is affordable economically and easily available. LMWH can be safely used in patients with severe acute pancreatitis with good clinical improvement.
